# Factors Affecting the Adherence to Partial Enteral Nutrition Combined with the Crohn’s Disease Exclusion Diet in Crohn’s Disease Adult Patients

**DOI:** 10.3390/healthcare14172674

**Published:** 2026-08-22

**Authors:** Vaios Svolos, Dimitra Eleftheria Strongylou, Georgios Charmantzis, Evgenia Popko, Dimitra Kanta, Christos Argyriadis, Dimitrios Grigoriou, Kalliopi Anna Poulia, Andreas Kapsoritakis, Konstantinos Argyriou, Odysseas Androutsos

**Affiliations:** 1 Lab of Clinical Nutrition and Dietetics, Department of Nutrition and Dietetics, School of Physical Education, Sports Science and Dietetics, University of Thessaly, 42100 Trikala, Greece; vaiossvolos@gmail.com (V.S.); ntemi_st@hotmail.com (D.E.S.); georgecharmantzis2@gmail.com (G.C.); evgepopko@gmail.com (E.P.); dimitra.kanta@gmail.com (D.K.); cargyriadis@uth.gr (C.A.); dimitrisgrigoriou3@hotmail.com (D.G.); 2 School of Medicine, Dentistry and Nursing, College of Medical, Veterinary and Life Sciences, University of Glasgow, Glasgow G12 8QQ, UK; 3 Laboratory of Dietetics and Quality of Life, Department of Food Science and Human Nutrition, Agricultural University of Athens, 11855 Athens, Greece; lpoulia@aua.gr; 4 Department of Gastroenterology, University Hospital of Larisa, 41100 Larisa, Greece; kapsoritakis@uth.gr (A.K.); koargyri@med.uth.gr (K.A.)

**Keywords:** Crohn’s disease, adherence, CDED, PEN, EEN, qualitative, factors

## Abstract

**Background/Objectives**: Enteral nutrition (EN), delivered either exclusively (EEN) or partially (PEN) in combination with the Crohn’s Disease Exclusion Diet (CDED), represents an evidence-based dietary therapy for active Crohn’s Disease (CD), recommended in clinical guidelines. However, adherence to this therapy remains suboptimal among adult patients. This study aimed to explore the intention to repeat CDED & PEN alongside perceived factors affecting adherence to CDED & PEN in CD adult patients. **Methods**: A single-center, cross-sectional qualitative study was conducted between October 2025 and March 2026 at a private dietetic practice in Larissa, Greece. Semi-structured interviews were undertaken and analyzed using thematic analysis. **Results**: Out of the 88 patients screened, 15 adults with CD participated in semi-structured interviews. Four overarching themes emerged: (1) intention to repeat CDED & PEN, with all participants reporting willingness to repeat CDED & PEN in the event of future relapse; (2) barriers to CDED & PEN adherence, including challenges in social situations involving food; (3) facilitators of CDED & PEN adherence, such as improved symptom control and increased sense of security; and (4) dual factors affecting CDED & PEN adherence, whereby meal preparation demands, taste and variety, and social and environmental support acted as either facilitators or barriers against adherence depending on individual circumstances. **Conclusions**: Greek adults with CD showed strong willingness to reinitiate CDED & PEN during relapse. Addressing modifiable barriers, particularly dietary monotony and financial burden, alongside strengthening structured dietitian support and personalized dietary guidance, may help optimize adherence in clinical practice.

## 1. Introduction

Crohn’s Disease (CD), alongside ulcerative colitis, constitutes a principal form of Inflammatory Bowel Disease (IBD) that affects the gastrointestinal (GI) tract. CD is a chronic condition characterized by transmural inflammation that can affect any part of the GI tract, from the mouth to the anus, most commonly involving the ileum and ascending colon. The disease follows a relapsing–remitting course and is associated with significant GI and systemic complications, contributing to substantial morbidity and reduced quality of life [1,2].

CD incidence and prevalence vary geographically, with the highest burden reported in Oceania, Europe, and North America [3,4,5,6]. Incidence reaches 29.3 cases per 100,000 person-years in Australia, while prevalence reaches 322 per 100,000 individuals in Germany [3,4]. Rates are also rising in newly industrialized countries, likely reflecting Westernized lifestyles [4,7]. CD affects both sexes and most commonly occurs between 15 and 35 years of age [1,6].

Environmental exposures, genetic susceptibility, immune dysregulation, and gut microbiota dysbiosis contribute to CD pathogenesis and disease course. Smoking doubles CD risk and is associated with a more severe disease phenotype [8,9], while antibiotic exposure may contribute through gut microbiota disruption and immune dysregulation [10,11]. Westernized diets high in refined sugars, saturated fats, and food additives may promote dysbiosis and inflammation [12,13], whereas higher intake of fiber, fruits, and vegetables may have protective effects [14].

Currently, there is no definitive cure for CD, and management strategies primarily involve a combination of pharmacological therapies and dietary interventions that induce and maintain remission, prevent disease progression, and contribute to improving patients’ quality of life [15,16,17]. Pharmacological treatment of CD follows a stepwise approach based on disease severity and complications [18]. Corticosteroids induce remission in active CD, while immunosuppressants, biologics, and antibiotics are used for maintenance or specific complications, with treatment tailored to disease location [19]. Current dietary therapies in CD include exclusive enteral nutrition (EEN), which is currently recommended for inducing remission according to the European Crohn’s and Colitis Organisation Consensus [20]. EEN helps induce and maintain remission, prevent symptom exacerbation and micronutrient deficiencies such as calcium, iron, zinc, and magnesium, modulate gut microbiota, and improve perioperative outcomes [21,22].

While EEN remains the preferred approach for inducing remission, its strict adherence can be challenging for many patients, leading to poor tolerance or adherence; in such cases, Partial Enteral Nutrition (PEN) or structured dietary interventions like the Crohn’s Disease Exclusion Diet (CDED) can be used as effective, more sustainable alternatives to support disease management [23,24,25,26,27]. CDED is a structured dietary intervention designed to reduce inflammation, improve gut dysbiosis, and support GI healing in CD [28,29]. Delivered in three phases, it begins with the exclusion of potential trigger foods while emphasizing high-quality protein and microbiome-supporting ingredients (weeks 0–6), followed by the gradual reintroduction of selected foods (weeks 6–12), and then a maintenance phase from week 13 onward that is personalized for long-term adherence. Often combined with PEN, CDED replaces pro-inflammatory components with anti-inflammatory foods and specialized formulas and is currently the only whole-food–inclusive dietary therapy with reproducible evidence for managing CD [30,31]. Notably, studies have shown that in both adult and pediatric patients with CD, CDED combined with PEN is better tolerated and equally effective compared with EEN in achieving clinical remission and reducing fecal calprotectin levels [32,33].

Although adherence to CDED & PEN in CD patients might be higher compared to EEN, adhering to these diets can still be challenging [32,34,35]. Factors such as lack of flexibility, social pressures and professional commitments can hinder strict adherence to these diets [34]. In addition, CD patients, when following restrictive diets may experience adverse social and psychological outcomes [36]. Evidence suggests that structured support and guidance from dietitians play a key role in facilitating adherence to therapeutic diets, thereby improving adherence among adults with CD [35,37].

However, although previous research has identified factors associated with dietary adherence, and a recent Dutch cohort study [37] has provided insights into patient experiences with CDED & PEN among children and adults, the lived experiences of adults following this dietary intervention warrant further in-depth qualitative exploration. In particular, the practical, social, and psychological challenges of implementing CDED & PEN, as well as the role of support, perceived benefits, and factors influencing willingness to repeat the intervention, remain insufficiently understood. Moreover, dietary practices and social eating are shaped by cultural context, highlighting the importance of exploring these experiences across different settings. Examining the experiences of adults with CD in Greece may therefore extend the existing evidence by providing insight into how CDED & PEN is experienced within a different social and cultural food context. Giving voice to patients and understanding their lived experiences can provide valuable insights for developing tailored approaches to support and enhance CDED & PEN adherence [38,39].

Therefore, this qualitative study aimed to explore in depth the experiences and perspectives of adults with CD who had previously followed CDED & PEN, with a particular focus on barriers and facilitators to adherence, the perceived impact of the intervention on daily life, and factors influencing willingness to repeat the dietary therapy.

## 2. Materials and Methods

### 2.1. Study Design

This study employed a cross-sectional qualitative design using semi-structured interviews and inductive thematic analysis to examine in depth the perceptions, experiences, perceived barriers, and facilitators regarding the implementation of CDED & PEN among adults with CD who had prior experience with this dietary therapy.

### 2.2. Ethical Approval

Ethical approval was obtained from the Ethics Committee of the Department of Nutrition & Dietetics, University of Thessaly (approval no: 49/28.05.2025). All study procedures were conducted in accordance with the institutional code of conduct, legal requirements, and established ethical guidelines defined by the University Ethics Committee. No adverse events were reported during or after the completion of the individual interviews, and the qualitative section was completed as planned.

### 2.3. Study Setting and Recruitment

Data collection took place between October 2025 and March 2026 at a private dietetic practice in Larissa, Greece. Adult patients with a confirmed diagnosis of CD were recruited through the private dietetic practice and invited to participate in the study. Although participants were recruited through the private dietetic practice, none had received their previous CDED & PEN treatment from the researchers conducting the interviews. Interested individuals completed a brief online screening questionnaire consisting of closed-ended items assessing sociodemographic and clinical characteristics, as well as prior use of dietary therapies. The questionnaire was used to identify individuals with prior experience of CDED & PEN who were eligible for participation in the interview study.

Patients who met the eligibility criteria (*n* = 20) were contacted by the research team via email and provided with a detailed participant information sheet outlining the study aims, procedures, potential risks and benefits, confidentiality measures, and the voluntary nature of participation. Of the 20 eligible individuals, 15 expressed interest in participating and completed the interviews, while 5 did not respond to the invitation. Although a purposive approach had been intended to ensure variation in participant characteristics, all 15 individuals who expressed interest were included in the qualitative study, and no further selection procedures were applied.

### 2.4. Eligibility Criteria

Inclusion criteria were: (1) age ≥ 18 years, (2) self-reported, confirmed diagnosis of CD, (3) residence in Greece, (4) ability to communicate in Greek, and (5) prior experience with the CDED & PEN regimen. All eligible individuals who provided informed consent were included in the study.

### 2.5. Data Collection

Semi-structured interviews were conducted online via Microsoft Teams at a time convenient for participants. Online delivery was selected to maximize accessibility and flexibility. Prior to each interview, written informed consent was confirmed electronically, and participants were given the opportunity to ask questions.

Interviews were conducted by members of the research team (G.C., BSc and D.E.S., PhD), who introduced the study and ensured participants were comfortable before proceeding. No other individuals were present during the interviews. Each participant completed one interview lasting approximately one hour. Participants were informed that they could pause or withdraw at any time without consequence; however, no withdrawals occurred. All interviews were conducted in Greek and audio-recorded with participants’ consent. Audio recording was used instead of video recording to minimize the collection of identifiable information and enhance participants’ privacy. Interviews were transcribed verbatim in Greek, and transcripts were checked against the audio recordings for accuracy. For reporting in the manuscript, selected quotations were translated from Greek into English. The English translations were subsequently back-translated into Greek and compared with the original quotations to ensure that the meaning and context were accurately preserved.

Interviews followed a semi-structured topic guide (Table 1) exploring participants’ experiences with the CDED & PEN, including perceived benefits and challenges, impact on physical and mental health, factors influencing adherence, and intentions regarding future use of dietary therapy. Probing questions were used to encourage depth and clarification of responses.

### 2.6. Materials

The screening online questionnaire consisted of structured, closed-ended questions and was organized into predefined sections to systematically capture participant information. These sections included: (1) sociodemographic characteristics (i.e., age, education level, employment status, and gender), (2) past treatment with CDED & PEN, and (3) disease-related information that encompassed disease duration since diagnosis, current pharmacological treatment and history of hospitalizations.

The interviews were conducted using a nine-question semi-structured topic guide (Table 1). Questions explored CD patients’ thoughts, beliefs, and perceptions regarding the impact of CDED & PEN in their lived experiences, physical and mental health, as well as their intention to repeat the dietary intervention. To encourage deeper reflection and clarify emerging topics, probing and follow-up questions were used. The semi-structured interview topic guide was piloted by independent gastroenterologists, dietitians, and psychologists, as well as two patients with Crohn’s disease who were not included in the study. Feedback was used to ensure the clarity, relevance, and appropriateness of the questions.

**Table 1:** Questions of the semi-structured topic guide.

Question 1. What was your experience with Partial Enteral Nutrition (PEN) in combination with the Crohn’s disease exclusion diet (CDED)? (For example, how did you find it? What do you remember most vividly from the period you followed it? How would you describe this period to someone else? When did you do it? Did you do it once? For how long? What other medication were you taking?)
Question 2. For how long did you follow it?
Question 3. What are your thoughts on possibly repeating it in a new flare-up period? (For example, would you do the same again? If yes, in what situation? If not, for what reason?)
Question 4. What was positive, that is, what did you like about this experience? (For example, what were the advantages? For example, some people report that it helps with Crohn’s symptoms. Others mention that they don’t have to worry about what they should eat.)
Question 5. What was negative, that is, what did you not like about this experience? (For example, what were the disadvantages? For example, some people report having difficulty when they need to attend a social event. Cost-Prescription, Cooking, Time, Social eating/eating out, Taste/pleasure)
Question 6. What helped you follow it? (For example, how did you decide to follow this plan; Role of the dietitian; Role of the doctor; Role of the family; Who cooked?; Recipes; Motivation; Benefit to symptoms)
Question 7. What made it difficult for you to follow it? (For example, social eating—Physical symptoms, e.g., constipation; Support from your environment; Cooking)
Question 8. If you were to follow this treatment again, what would you change to make it easier to follow?
Question 9. Would you try, in a possible future flare-up, to follow exclusive enteral nutrition (consuming only powdered milk)?

### 2.7. Data Analysis

A four-step thematic analysis following Braun and Clarke [40] was conducted to analyze the interview data using an inductive approach. The four stages of analysis encompassed: (1) Initial data familiarization: transcripts were read multiple times and initial thoughts were recorded. (2) Generation of initial codes: the transcripts were systematically examined and segments of data relevant to the research questions and participants’ experiences of CDED & PEN were coded. Codes captured meaningful features of the data, including participants’ perceptions, experiences, barriers, facilitators, and views regarding future use of the dietary intervention. The initial codes were developed directly from the interview data and were iteratively refined during the coding process as similarities and differences across transcripts were identified. Coding was primarily semantic, focusing on participants’ explicit accounts and meanings and was conducted across all transcripts to identify patterns and similarities within the dataset. (3) Searching for themes: codes with related meanings were compared and grouped into candidate themes, and relationships between codes were considered to identify broader patterns of meaning across participants. (4) Reviewing themes: candidate themes were reviewed against the coded extracts and the dataset as a whole and were refined, combined, or reorganized where appropriate. The final themes were subsequently defined and named according to their central organizing concept. It should be noted that as all 15 eligible participants who expressed interest in the study were interviewed, no further recruitment attempts were made. Retrospectively, the final interviews did not yield substantively new patterns or themes beyond those already identified in the dataset. Field notes taken during the interviews were also reviewed and considered during the analysis to provide additional contextual information.

The interviews were conducted by two colleague researchers, one female health psychologist (D.E.S.) and one male dietitian (G.C.), who had different levels of previous experience in qualitative interviewing. The dietitian had prior clinical experience with dietary management in Crohn’s disease, including CDED & PEN, whereas the health psychologist had expertise in psychological and behavioral aspects of chronic disease management. Neither interviewer was directly involved in the clinical management or previous CDED & PEN treatment of the interviewees. This separation from participants’ clinical care was intended to minimize potential power dynamics and encourage participants to speak openly about their experiences, including difficulties and negative perceptions of the dietary intervention. The researchers recognized that their professional backgrounds, experience, and perspectives could influence both the interview process and interpretation of the data. These differences were therefore considered during the analysis. The two researchers independently coded the data and reviewed the identified themes, using their differing professional perspectives to critically discuss the interpretation of the data. Discrepancies were explored through discussion and resolved by consensus.

NVivo (version 14) software (Lumivero, Denver, CO, USA) was used to support the organization and management of the dataset, codes, and emerging themes during the analytical process. The reporting of the qualitative study was guided by the Consolidated Criteria for Reporting Qualitative Research (COREQ) checklist [41], which is provided as Supplementary Material (please see Table S1).

## 3. Results

### 3.1. Participant Characteristics

Of the 88 patients who completed the screening questionnaire, 20 (22.8%) reported previous experience with CDED & PEN during a disease exacerbation and were therefore eligible for the qualitative interview study. Of these 20 eligible individuals, 15 expressed interest in participating and completed the interviews, while 5 did not respond to the invitation. All 15 participants who expressed interest were included in the qualitative study, and no further selection procedures were applied. In those 15 participants the median age was 36.5 years. Disease duration varied across participants, ranging from less than 1 year to more than 10 years. The study included 6 males (40.0%) and 9 females (60.0%). Regarding educational level, most participants had completed university or postgraduate education, except for the youngest participant, who had completed high school. Most participants were employed full-time or part-time, and 66.7% reported previous CD-related hospitalization. Biological agents were the most commonly reported pharmacological treatment (73.3%), followed by corticosteroids (20.0%), immunosuppressants (13.3%), and other treatments. Participant characteristics are presented in Table 2.

### 3.2. Themes

**Figure 1:**
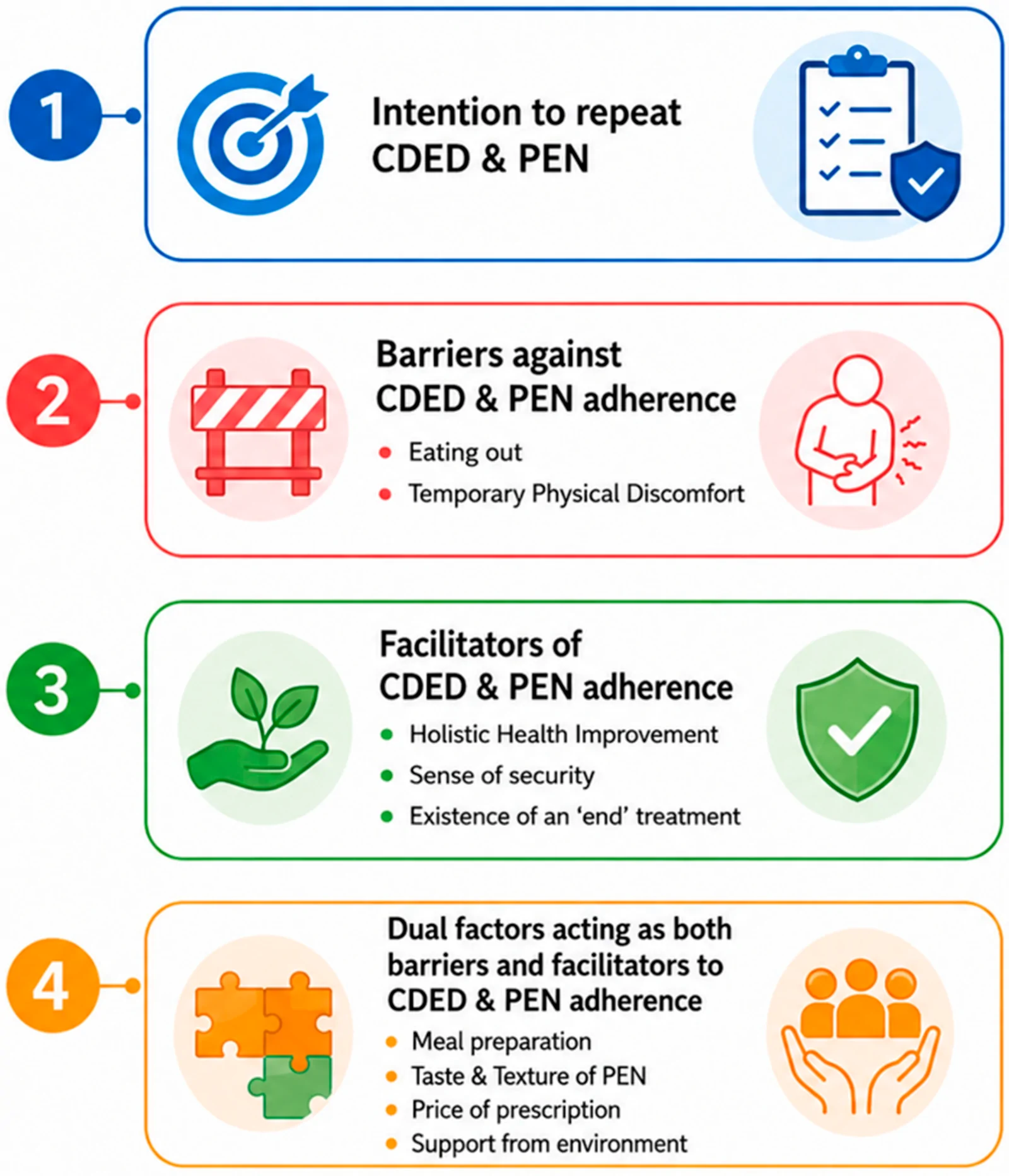
Themes emerged from the implementation of CDED & PEN: (1) “Intention to repeat CDED & PEN”, (2) “Barriers against CDED & PEN adherence”, (3) “Facilitators of CDED & PEN adherence” and (4) “Dual factors acting as both barriers and facilitators to CDED & PEN adherence”.

Four key themes emerged from the analysis regarding the impact of the dietary regimen. Concerning the first theme, “intention to repeat CDED & PEN”, all participants expressed willingness to repeat the CDED & PEN during future disease flare-ups, indicating high overall acceptability of the intervention. With regard to “barriers against CDED & PEN adherence”, the main barriers that emerged concerned difficulties in eating out for most interviewees, while some experienced physical discomfort and symptoms, such as diarrhea, constipation, or bloating, during the first 1–2 weeks of implementing CDED & PEN. Factors identified under the theme “facilitators of CDED & PEN adherence” included positive physical and mental effects during relapse, a sense of security, and the presence of a clearly defined treatment endpoint, all of which positively influenced patient adherence. The “dual factors acting both as barriers and facilitators to CDED & PEN adherence” theme demonstrates that factors dependent on individual and cultural circumstances, such as meal planning, cost, and support, can act as both facilitators and barriers. An overview of the key themes is shown in Figure 1, and each theme is separately and in-depth described below. It should be noted that data were not sub-analyzed according to key demographic or clinical characteristics. However, where relevant differences or patterns emerged during the analysis, these were noted and presented within the corresponding themes. Otherwise, findings are reported across the entire sample.

#### 3.2.1. Theme 1: Intention to Repeat CDED & PEN

Overall, all interviewees expressed a willingness to repeat CDED & PEN. More specifically, all participants indicated that in the event of a future relapse, they would follow the CDED in combination with PEN, as they had previously experienced symptom improvement with this approach.

*“I would do it again, yes, yes, if it worked the first time, I would do it again.”*

*(QPEN12, Female)*

*“But if this was the catalytic factor for the body to recover, yes, of course. With absolute certainty.”*

*(QPEN09, Female)*

Furthermore, all participants reported that, if required to alleviate their symptoms, they would even be willing to attempt even EEN. One participant had previously undergone EEN on two occasions for the treatment of a perianal fistula. Although the fistula did not improve, the participant reported well-controlled bowel movements during EEN, describing them as “as if there were no Crohn’s disease”.

*“I consumed polymeric feed for 175 days… one 100 days, the other 175 days without any other food intake, not even half a bite of any food. During this time, let’s say for the 175 days I did it in an attempt to close the perianal fistula that I had been active for years… Perfect bowel movements as if you didn’t have Crohn’s. Unfortunately, however, I was unable to treat the perianal fistula.”*

*(QPEN11, Male)*

The same participant, who had experience with both CDED & PEN and EEN, reported comparable positive effects in terms of symptom control and overall disease management:

*“As a result, I can say that both—CDED & PEN and EEN—were equally good.”*

*(QPEN11, Male)*

#### 3.2.2. Theme 2: Barriers Against CDED & PEN Adherence

The barriers identified in the analysis were primarily individual in nature and included challenges related to managing social eating situations, communicating dietary requirements to others, and coordinating transportation or travel to prepare and consume meals. These barriers often led to reduced flexibility in daily life and, in some cases, compromised adherence to the dietary combination.

Many participants reported difficulty in eating meals outside the home, as appropriate meal options were limited in restaurants. As a result, participants described either abstaining from eating in these contexts or preparing and bringing their own meals. These limitations were perceived as restrictive and, in some cases, reduced participants’ ability to fully engage in social activities. This restriction in social settings occasionally led to decreased adherence, especially when participants were unable or struggled to follow the dietary requirements outside the home. One participant described how this affected participation in social events and required ongoing preparation:

*“It was a bit restrictive in the sense that I could not easily participate in anything that might… Let’s say we had a party in a group, and all of that I might not drink, or I could not try everything, usually I was prepared with my own things to eat at work, or out on a walk, or whatever. At first, this limited me a bit and really distracted me because I had to spend a lot of time preparing everything, but in the process, I got used to it very easily.”*

*(QPEN08, Female)*

In addition to practical challenges related to food availability, some participants reported difficulties communicating their dietary practices to friends and relatives. These difficulties stemmed from concerns about unsolicited comments, questioning, or criticism, which participants described as emotionally taxing alongside the demands of adhering to dietary restrictions. Several participants indicated that this social pressure negatively affected their ability to maintain the diet, particularly in social settings where they felt scrutinized or compelled to justify their food choices. As a result, feelings of stress, discomfort, and discouragement were reported as barriers to adherence. One participant reflected on the challenge of explaining their dietary practices and managing others’ reactions. This emotional burden negatively affected adherence for some participants by increasing stress and making it more difficult to maintain the dietary regimen in social situations.

*“But if you look at it more openly, yes, it is annoying, it is annoying even to explain to the other person why you don’t eat like that. Or why do you have to eat what you eat or are you sure that what you are doing is right? You see that in the end you are not doing it right and this whole story of everyone saying whatever they want as they want, which okay, in part, can be (…) someone is right, just when you are in a difficult position, it is difficult to listen to them.”*

*(QPEN12, Female)*

A few participants reported difficulties related to mobility and travel, which were attributed to the need to carry polymeric feed and to secure access to safe food choices outside the home. These challenges also affected traveling due to logistics, which in some cases reduced adherence, particularly when participants were unable to maintain the dietary regimen while away from home. Participants described the need for careful planning, explaining how polymeric feed had to be consumed on the move.

*“It was to a certain extent -difficult-, that is, think about it, I had gone on a trip that Christmas when I started it, abroad. And I had to carry polymeric feed with me. I would take a water bottle with me and drink, in a phase.”*

*(QPEN03, Male)*

As one participant further explained, he had to adjust his daily activities and constantly adapt his routine based on the consumption of polymeric feed. This need for constant routine adjustment made adherence more difficult in daily life, particularly when flexibility was required.

*“It was difficult for me, the truth is because “I was forced” to come home earlier or to come home -a rented room- and then go out again so I could eat my meals or drink polymeric feed.”*

*(QPEN02, Male)*

A few participants mentioned experiencing temporary adverse effects, such as constipation, bloating, nausea, metallic taste, and tingling sensations during the first days or weeks after the implementation of treatment. These participants emphasized that these symptoms were short-term and lasted as the body adapted to the new dietary therapy. Although subtle, these symptoms initially hindered patients’ adherence:

*“Just then, because I was drinking a lot of polymeric feed, okay, it would come and make me very angry (…) and around evening I noticed that the last polymeric feed caused a strange taste in my mouth and a tingling sensation. However, okay, it didn’t last more than two weeks, then I got used to it.”*

*(QPEN04, Female)*

#### 3.2.3. Theme 3: Facilitators of CDED & PEN Adherence

Participants identified several factors that facilitated adherence to CDED & PEN treatment, including holistic health improvement, symptom reduction, psychological improvement, and a sense of security. In addition, having a clearly defined goal and a structured schedule was described as supportive of maintaining adherence to the dietary regimen.

In particular, most participants reported marked improvements in their clinical condition following the implementation of PEN combined with CDED. Patients experienced a reduction in the frequency of bowel movements, improvement in stool consistency, as well as a significant decrease in pain and other GI symptoms. These perceived improvements acted as a strong motivating factor for continued adherence to the treatment.

*“Yes, I saw a difference in toilet visits as well; they decreased. I had reached a point where I had gone from 10 to the peak, let’s say, it was one day, I had gone 10 times, I had fallen to 3, then another positive thing was that the pain decreased. Slowly, the pain subsided as well.”*

*(QPEN13, Female)*

Improvements in stool consistency were also highlighted, with participants describing stools as more formed and less watery. Additionally, some participants noted stabilization or restoration of body weight, which was perceived positively. These improvements reinforced adherence, as participants associated the dietary regimen with tangible health benefits.

*“Well, they were more formed, they weren’t so watery anymore, and that was a positive thing that I remember.”*

*(QPEN07, Female)*

*“Well, I wasn’t losing weight either…”*

*(QPEN10, Female)*

Additionally, several participants reported improvements in their psychological well-being, noting a reduction in anxiety regarding disease course and a sense of security provided by the dietary regimen in the event of future exacerbation. This psychological reassurance facilitated adherence by reducing fear and uncertainty related to disease management.

*“Well, from the moment everything worked and went well, okay, the anxiety went away, and then there was, you know, hope.”*

*(QPEN04, Female)*

The rapid onset of symptom relief further supported adherence by strengthening participants’ motivation to continue with CDED & PEN. These included a significant reduction or complete resolution of pain, improved bowel habits, maintenance or restoration of a healthy body weight, and increased energy and strength:

*“It helped me with the pain and bowel movements.”*

*(QPEN06, Female)*

*“Um, that you were gaining weight in a very short period of time, which you have lost sharply in the past.”*

*(QPEN02, Male)*

One participant also reported a notable clinical improvement following the implementation of the dietary regimen, describing the resolution of a previously identified stenosis. This improvement was perceived as unexpected and was attributed, at least in part, to the introduction of CDED & PEN alongside existing treatment:

*“Well, when we went and saw the point again, at the point where we had the wounds and the stenosis, because a stenosis always scares us. And even the doctor himself cannot answer what happened there, and the stenosis was not there. Did it all help? The biologic that you were taking anyway helped, so the biologic alone cannot have helped the stenosis because the stenosis was there, because I have been taking the biologic for 10 years. So, what did we do now? We did a small amount of cortisone and the supplement of polymeric feed. So, what happened there now? There is no stenosis. We clarify that there is no stenosis. And the wounds we can say were at the same level, slightly better.”*

*(QPEN15, Male)*

Most participants stated that following a structured nutritional regimen contributed to a sense of safety and trust in the treatment. They described feeling safer in their dietary choices and less concerned about triggering symptoms, which reduced fears related to eating and disease exacerbation. This sense of control facilitated adherence by reducing uncertainty around food choices and symptom triggers. Participants revealed that they felt more confident in managing their condition throughout their treatment:

*“I would definitely put on the positive side the fact that I felt completely safe in my diet, that is, I would not be afraid that I would get cramps again or get stuck, some food in the stricture and need to vomit again or feel pain again or anything like that, and I would definitely put on the positive side the fact that I felt myself and much cleaner I would say. And that’s what helped me so much to set limits on myself.”*

*(QPEN01, Female)*

A few participants highlighted the importance of adherence to nutritional therapy for a specific time. The presence of a defined duration and structured timeline facilitated adherence, as participants found it easier to comply with dietary restrictions when they were aware that the regimen would eventually be completed. This expectation provided reassurance and motivation, particularly in relation to the possibility of reintroducing previously restricted foods.

*“And my nutritionist told me after a few weeks or months, I don’t remember, he told me, I know, you can slowly incorporate some things into your diet plan, so that you can get away from chicken a little. That helped me, yes.”*

*(QPEN02, Male)*

#### 3.2.4. Theme 4: Dual Factors Acting as Both Barriers and Facilitators to CDED & PEN Adherence

Several factors were found to have a dual role in participants’ experiences with CDED & PEN treatment, functioning as facilitators for some patients and as barriers for others. These factors were related to practical, sensory, financial and social aspects of treatment implementation, and their influence depended on individuals’ circumstances and support systems. Namely, these factors were meal preparation and planning, sensory (including taste, variety, and monotony of foods), price of PEN subscription, and social support.

Changes in eating habits and meal preparation emerged as a factor that both facilitated and challenged adherence to the dietary regimen. In particular, about half of the participants described meal preparation as a facilitating factor. The reduced cooking time due to the simplicity of the permitted foods acted as a facilitating factor and enhanced adherence to the treatment:

*“Compared to before I did this diet, I devoted less time to cooking because it was quite simple.”*

*(QPEN07, Male)*

On the contrary, for the remaining half participants, the need to plan meals and prepare separate food independently was perceived as time-consuming and required more effort, acting as a barrier to treatment adherence. As some participants explained, the time-consuming preparation of separate meals by the family also limited the ease of adherence, particularly during busy schedules or work-related constraints.

*“It tires me a bit—preparing meals—and because of work hours. Because I had to prepare everything myself, let’s say if I was at work I might eat the staff’s food.”*

*(QPEN08, Female)*

*“Uh, otherwise, if we enter this process, of course, it takes a lot more time, it requires other preparation. Okay, some foods can be eaten, and the rest is not something tragic, but let’s say, I couldn’t eat moussaka while the others were eating.”*

*(QPEN12, Female)*

Most participants reported being satisfied with the taste of both the polymeric feed and the permitted foods included in the dietary regimen, which was perceived as an important facilitator of adherence. Positive perceptions of palatability appeared to support sustained engagement with the diet and reduce challenges associated with dietary restriction. One participant described the polymeric feed in particularly favourable terms:

*“It seemed to me the most delicious thing on earth, and this milk, along with the cocoa powder that I usually drank, seemed to me the sweetest and most perfect, er, chocolate milk that I could drink at that moment.”*

*(QPEN01, Female)*

However, a smaller number of participants reported challenges related to the taste and texture of the polymeric feed, particularly its sweetness and thickness, which made adherence more difficult during the initial stages of the treatment.

*“Sometimes I had a hard time and didn’t take all the doses, maybe I skipped one. Yes, yes, yes, yes, I mean, I had drunk too much, so I couldn’t stand it. I didn’t want to drink any more -because of the taste.—How would you characterize this taste?— (…) Well, its texture was a bit thick, I should say?”*

*(QPEN07, Male)*

For some participants, limited food variety aligned with their usual eating habits and personal preferences, thereby facilitating adherence:

*“I generally don’t have great variety in food; I eat specific things, so this helped me even more in following the diet.”*

*(QPEN02, Male)*

In contrast, almost half of the participants expressed negative feelings about the exclusion of favorite foods, while many participants reported limited variety and repetitiveness in the permitted foods, factors that negatively affected adherence:

*“Uh, the truth is that it was a little, it was a little tough, especially after 3 weeks, because the foods were very limited.”*

*(QPEN05, Male)*

*“But at the beginning, we would start with the classic chicken with baked potatoes every day. At some point, I said, ‘Oh, chicken again, baked potatoes.”*

*(QPEN11, Male)*

About half of the participants reported that the cost of polymeric feed and challenges related to prescription coverage negatively influenced adherence. Participants described the financial burden associated with purchasing enteral nutrition, particularly when prescriptions were not approved or required frequent renewal:

*“The preparations are very expensive. The state did not accept the recommendation that had been issued by the city’s public hospital.”*

*(QPEN10, Female)*

It was also reported that when polymeric feed was successfully prescribed and subsidized, the cost was considered manageable and facilitated adherence:

*“In terms of cost, when you had a prescription, the 10 boxes were available at an affordable price.”*

*(QPEN03, Male)*

Most participants mentioned that the support from healthcare professionals and/or their personal environment was a decisive facilitator of adherence. Participants emphasized the importance of encouragement, trust, and positive attitudes from dietitians and physicians:

*“I got very positive vibes from my dietitian and very, very positive motivation in terms of his trust in the whole process-diet.”*

*(QPEN08, Female)*

*“I also had this support with my family, and I was, as they say, glowing”*

*(QPEN10, Female)*

At the same time, as most participants explained, the provision of alternative recipes and the dietary flexibility offered by specialized dietitians significantly facilitated adherence:

*“He gave very nice recipes, which I… I told you, I could stay with a little, and I tried several of them that worked for me…”*

*(QPEN13, Female)*

On the contrary, it is certainly worth noting that a few participants described the intense feeling of loneliness that they experienced due to the lack of support from their personal environment and the medical staff, resulting in difficulties in adherence to the treatment:

*“A very important factor that pushes a person with Crohn’s to isolation… and not to have it.”*

*(QPEN01, Female)*

*“She believed—the doctor—that you can eat everything in moderation; this in itself did not make it easier at all… but I would also like her trust in all this.”*

*(QPEN08, Female)*

## 4. Discussion

This cross-sectional qualitative study provides an exploration of CD patients’ experiences of dietary therapies, with particular emphasis on factors influencing adherence to the combination of CDED & PEN. Overall, findings indicate high intention to repeat the CDED & PEN dietary intervention, with participants expressing a clear willingness to reinitiate treatment in the event of symptom relapse. The findings of this study are consistent with, and extend, existing evidence regarding the feasibility and acceptability of CDED-based dietary interventions in CD adult patients [31,37,42,43].

The strong intention to repeat CDED combined with PEN observed in the present study is consistent with previous evidence from both pediatric and adult populations, which suggests that this approach is generally well tolerated and often preferred over more restrictive nutritional therapies [32,44].

In our cohort, participants’ willingness to undertake the intervention again appeared to be driven largely by their perceived symptom improvement and confidence in its therapeutic effectiveness, underscoring the importance of positive treatment experiences in shaping future treatment preferences. This suggests that patient acceptability is influenced not only by clinical outcomes but also by how manageable and worthwhile the intervention is perceived to be.

These findings are consistent with those reported by Levine et al., who demonstrated in a pediatric randomized controlled trial that CDED combined with PEN was comparable to EEN in maintaining remission while offering greater tolerability and acceptability [32]. The authors proposed that permitting the consumption of selected solid foods may lessen the burden associated with dietary therapy and support sustained adherence. Further evidence comes from Wijers et al., who reported high levels of treatment satisfaction and willingness to repeat CDED among both pediatric and adult patients, particularly when the intervention was accompanied by structured dietetic support and digital monitoring tools [37]. Collectively, these findings suggest that patient acceptability extends beyond perceived efficacy alone and is strongly influenced by the practical experience of treatment and the availability of ongoing support throughout the dietary intervention.

Beyond improvements in symptom burden, interviewees identified the structured and limited nature of the CDED & PEN programme as an important facilitator of adherence. Participants described a sense of reassurance in knowing that dietary restrictions would gradually ease over time, which appeared to enhance motivation and make the intervention feel more manageable. This finding is consistent with a Dutch real-world study of children and adults with mild-to-moderate CD, which reported generally positive experiences with CDED & PEN and found that many participants would recommend or repeat the intervention [37]. Notably, patients valued the accompanying Modulife support platform, particularly its clear dietary guidance and recipe resources, suggesting that structured support may help patients navigate the complexity of the diet and maintain adherence [37]. Together, these findings highlight the importance of predictability and structure in facilitating engagement with dietary therapy.

On the other hand, participants described how restricted restaurant options directly reduced adherence, particularly in contexts where eating out carries social and cultural significance. This mirrors findings from the Dutch cohort, where a quarter of participants reported difficulty bringing appropriate meals to school or work, especially during the less restrictive Phase II of CDED & PEN, whereas participants reported that it was harder to maintain [37]. A less common but notable barrier was transient physical discomfort during the first one to two weeks of the regimen, including constipation, bloating, nausea, metallic taste, and tingling sensations. Although no published study has specifically characterised an adaptation period with transient physical symptoms at the onset of CDED & PEN, early intolerance-related dropout is documented in the available literature [32,43]. The transient physical discomfort reported by participants in the present study may represent an early adaptation response before any perceived therapeutic benefit, and which is not yet captured by quantitative study designs. This qualitative insight suggests that proactively informing patients about the possibility of early symptoms prior to treatment initiation may be an important strategy for preventing dropout during the most critical phase of the regimen.

Meal preparation and sensory perception of PEN functioned as bidirectional factors, depending on individual circumstance rather than on the regimen itself. This is consistent with broader IBD literature identifying work schedules and food-related routines as context-dependent determinants of dietary adherence [36]. Perceptions of the polymeric formula’s taste and texture were similarly variable, with some participants enhancing the flavor with additives and others finding it too sweet or thick. While adults with CD generally prefer polymeric over elemental formula [45], this preference does not reliably predict adherence: pediatric data show comparable non-completion rates regardless of formula type [46]. Formula type appears less important than individual sensory tolerance and it is better addressed through flexible flavoring options than through formula selection alone. Cultural adaptation with Greek culinary traditions is similarly addressable. CDED has been successfully adapted to Australian, Slovenian, and Nordic food cultures while preserving its core exclusion principles [47,48,49,50], supporting dietitian-guided adaptation over protocol adherence. Collectively, these findings indicate that adherence depends less on the dietary rules themselves than on how well the regimen is tailored to each patient’s daily life.

The cost of PEN emerged as an important determinant of adherence, acting as either a barrier or facilitator depending on prescription coverage. For many participants, difficulties obtaining approval and the ongoing expense of purchasing PEN created a substantial financial burden that negatively affected adherence. In contrast, those with subsidised prescriptions generally reported that the cost was manageable and did not hinder continuation of the regimen. Similar findings have been reported in pediatric populations, where the financial burden of CDED & PEN was identified as a key barrier to adherence [35]. More broadly, reimbursement policies and insurance coverage have been shown to influence access to EN therapies and are recognized as important determinants of treatment uptake and adherence across healthcare systems [51]. These findings reinforce the importance of accessible prescription pathways in supporting real-world adherence to CDED & PEN.

Beyond financial accessibility, interpersonal and professional support emerged as a further determinant of adherence. All participants identified support from healthcare professionals, and particularly dietitians, and family as a meaningful contributor to adherence and a positive treatment experience. Conversely, a small number who experienced limited social support or felt isolated described reduced motivation and poorer adherence, underscoring that the absence of support is not a neutral condition but an active barrier. The centrality of dietitian input for ongoing monitoring, individualized guidance, and culturally adapted meal planning is consistent with wider evidence identifying multidisciplinary support as a key determinant of dietary adherence in CD [34,36,52].

An additional consideration is the suitability of restrictive dietary interventions for patients who may be at increased risk of disordered eating. Although this was not specifically assessed in the present study, the restrictive nature of CDED & PEN highlights the importance of careful patient selection and individualized dietetic support. Furthermore, given the financial burden associated with PEN reported by some participants, the overall burden and cost of CDED & PEN should be considered alongside its potential benefits and compared with other available therapeutic options. Future studies should therefore evaluate both eating-related vulnerability and the relative cost and burden of dietary therapy when assessing its long-term feasibility and acceptability.

This study has several strengths. Qualitative design is the most in-depth approach to capturing patients’ experiences with CDED & PEN. It reveals adherence factors that clinical outcome measures alone cannot capture, such as financial burden, taste tolerance, and the need for dietitian support. These are precisely the factors that determine whether a regimen can be delivered successfully in routine practice, not just whether it works biologically. The in-depth interviews generated rich and detailed accounts of participants’ experiences, allowing key barriers and facilitators to CDED & PEN adherence to be explored in depth.

However, several limitations should be acknowledged. The study was conducted in a single, relatively small urban setting, which may limit generalizability to other countries with different healthcare access or sociocultural settings. Although both genders were included, the study was not designed to formally examine gender-related differences in dietary practices or food-related responsibilities. As with all qualitative research, findings are subject to interpretative bias. To mitigate this limitation, the analysis was enhanced through independent coding by two researchers, with discrepancies resolved through consensus. Additionally, the interviews relied on self-reported data, which may be subject to recall and social desirability bias. This may undermine the exact phase that different factors affect adherence, as for example the restriction of foods is stricter in the first phase and more support or personalized guidance may be needed. The use of online interviews may have introduced selection bias, potentially excluding individuals with limited digital access. Furthermore, self-selection bias cannot be excluded, as 15 of the 20 eligible patients participated, while 5 did not respond. Participants may have been more motivated or positively disposed toward CDED & PEN, potentially contributing to the uniformly reported willingness to repeat the intervention and limiting transferability to patients with less favorable experiences. Recruitment through a private dietetic practice may also have favored individuals who were more engaged with nutritional management or had a particularly positive relationship with dietetic support. Because detailed information on participants’ previous CDED & PEN exposure, including duration, completion of the recommended phases, and any modifications or premature discontinuation, was not systematically collected, we cannot assess how differences in prior treatment experience may have influenced perceptions of feasibility, barriers, or willingness to repeat the dietary therapy. Future studies should examine these aspects in greater depth to better understand their influence on implementation and adherence.

These findings indicate that the real-world feasibility of CDED & PEN depends substantially on patient adherence, which is shaped by individual, financial, social, and cultural factors alongside perceived clinical response. Successful implementation therefore requires more than prescribing a dietary protocol; it necessitates access to trained dietitians who can provide structured, culturally sensitive, and ongoing support, help patients navigate social eating situations, and proactively address anticipated barriers to adherence. The findings also highlight the importance of accessible prescription pathways for PEN and suggest that digital support tools may further enhance patient engagement. From a research perspective, future studies should move beyond measuring adherence as a binary outcome and investigate the mechanisms that underpin successful engagement with dietary therapy, including the roles of dietetic support, social support, reimbursement policies, cultural adaptation, and digital interventions. Longitudinal mixed-methods studies are warranted to explore how adherence barriers and facilitators evolve across different phases of CDED & PEN, while economic evaluations should assess whether improving access to subsidised PEN can enhance adherence and clinical outcomes. Finally, the transient physical symptoms reported during the first weeks of treatment represent a potentially important and underexplored adaptation period that warrants prospective investigation as a target for early dropout prevention strategies.

## 5. Conclusions

This is the first qualitative study to explore the lived experiences of Greek adults with CD receiving CDED & PEN, focusing on the factors shaping real-world implementation. The findings demonstrate a strong intention to repeat the intervention, with adherence shaped by a complex interplay of symptom response, social context, financial accessibility, and dietitian-led support. Collectively, these results indicate that successful implementation of CDED & PEN extends beyond clinical efficacy and requires personalized, patient-centered dietary care integrated into routine clinical practice.

## Figures and Tables

**Table 2:** Characteristics of CD patients who participated in individual interviews (*N* = 15).

Variable	*n* (%) ^1^
Age (years), median (min–max)	36.5 (19.0–53.0)
Gender	
Male	6 (40.0%)
Female	9 (60.0%)
Diagnosis Duration	
>10 years	5 (33.3%)
6–10 years	1 (6.7%)
1–5 years	4 (26.7%)
7–12 months	5 (33.3%)
Previous Hospitalization	
Yes	10 (66.7%)
No	5 (33.3%)
Level of education	
Postgraduate studies	2 (13.3%)
University/Postsecondary education	12 (80.0%)
High school/Junior high school	1 (6.7%)
Employment	
Part-time Employed	3 (20.0%)
Full-time Employed	11 (73.3%)
Students	1 (6.7%)
Type of pharmacological treatment ^2^	
Biological agent	11 (73.3%)
Corticosteroids	3 (20.0%)
Immunosuppressants	2 (13.3%)
Other treatment	3 (20.0%)

^1^ *n* = number; % = percentage; ^2^ participants could receive more than one therapy; therefore, percentages do not total 100%.

## Data Availability

The datasets used and/or analyzed during the current study are available from the corresponding author on reasonable request. The following email address will be requested: oandroutsos@uth.gr.

## Supplementary Materials

The following supporting information can be downloaded at https://www.mdpi.com/article/10.3390/healthcare14172674/s1, Table S1: Consolidated criteria for reporting qualitative research (COREQ): a 32-item checklist for interviews and focus groups.

## Abbreviations

The following abbreviations are used in this manuscript:

CD	Crohn’s Disease
CDED	Crohn’s Disease Exclusion Diet
COREQ	Consolidated Criteria for Reporting Qualitative Research
EEN	Exclusive Enteral Nutrition
EN	Enteral Nutrition
GI	Gastrointestinal
IBD	Inflammatory Bowel Disease
PEN	Partial Enteral Nutrition
