# Supplementary material for: Factors Affecting the Adherence to Partial Enteral Nutrition Combined with the Crohn’s Disease Exclusion Diet in Crohn’s Disease Adult Patients
Source: Healthcare (Basel). 2026 Aug 22;14(17):2674. doi: 10.3390/healthcare14172674 (PMC13564709; doi:10.3390/healthcare14172674)
Supplement: Supplementary file 1 [file healthcare-14-02674-s001.zip › healthcare-4470243-supplementary.pdf]

Table S1: "Consolidated criteria for reporting qualitative research (COREQ): a 32-item checklist for interviews and focus groups"

Developed from: Tong, A.; Sainsbury, P.; Craig, J. Consolidated criteria for reporting qualitative research (COREQ): a 32-item checklist for interviews and focus groups. Int. J. Qual. Health Care 2007, 19, 349–357.

No.	Item	Guide questions / description	Reported on page / line no.
**Domain 1: Research team and reflexivity**			
Personal characteristics			
1	Interviewer / facilitator	Which author/s conducted the interview or focus group?	Yes, Page 4
2	Credentials	What were the researcher's credentials? E.g. PhD, MD	Yes, Page 4
3	Occupation	What was their occupation at the time of the study?	Yes, Page 6
4	Gender	Was the researcher male or female?	Yes, Page 6
5	Experience and training	What experience or training did the researcher have?	Yes, Page 6
Relationship with participants			
6	Relationship established	Was a relationship established prior to study commencement?	Yes, Pages 3, 4, 6
7	Participant knowledge of the interviewer	What did the participants know about the researcher? E.g. personal goals, reasons for doing the research	No
8	Interviewer characteristics	What characteristics were reported about the interviewer / facilitator? E.g. bias, assumptions, reasons and interests in the research topic	Yes, Page 6
**Domain 2: Study design**			
Theoretical framework			
9	Methodological orientation and theory	What methodological orientation was stated to underpin the study? E.g. grounded theory, discourse analysis, ethnography, phenomenology, content analysis	Yes, Page 5
Participant selection			
10	Sampling	How were participants selected? E.g. purposive, convenience, consecutive, snowball	Yes, Pages 3-4
11	Method of approach	How were participants approached? E.g. face-to-face, telephone, mail, email	Yes, Page 4
12	Sample size	How many participants were in the study?	Yes, Page 6
13	Non-participation	How many people refused to participate or dropped out? Reasons?	Yes, Pages 4 & 6
Setting			
14	Setting of data collection	Where was the data collected? E.g. home, clinic, workplace	Yes, Page 3
15	Presence of non-participants	Was anyone else present besides the participants and researchers?	Yes, Page 4

No.	Item	Guide questions / description	Reported on page / line no.
16	Description of sample	What are the important characteristics of the sample? E.g. demographic data, date	Yes, Pages 6-7
Data collection			
17	Interview guide	Were questions, prompts, guides provided by the authors? Was it pilot tested?	Yes, Pages 4-5
18	Repeat interviews	Were repeat interviews carried out? If yes, how many?	No
19	Audio / visual recording	Did the research use audio or visual recording to collect the data?	Yes, Page 4
20	Field notes	Were field notes made during and / or after the interview or focus group?	Yes, Pages 5-6
21	Duration	What was the duration of the interviews or focus group?	Yes, Page 4
22	Data saturation	Was data saturation discussed?	Yes, Pages 5-6
23	Transcripts returned	Were transcripts returned to participants for comment and / or correction?	No
**Domain 3: Analysis and findings**			
Data analysis			
24	Number of data coders	How many data coders coded the data?	Yes, Page 6
25	Description of the coding tree	Did authors provide a description of the coding tree?	No
26	Derivation of themes	Were themes identified in advance or derived from the data?	Yes, Page 5
27	Software	What software, if applicable, was used to manage the data?	Yes, Page 6
28	Participant checking	Did participants provide feedback on the findings?	No
Reporting			
29	Quotations presented	Were participant quotations presented to illustrate the themes / findings? Was each quotation identified? E.g. participant number	Yes, Pages 8-13
30	Data and findings consistent	Was there consistency between the data presented and the findings?	Yes, Pages 8-13
31	Clarity of major themes	Were major themes clearly presented in the findings?	Yes, Pages 8-13
32	Clarity of minor themes	Is there a description of diverse cases or discussion of minor themes?	Yes, Pages 8-13
